# Supplementary material for: Guideline Concordance of Statin Treatment Decisions: A Retrospective Cohort Study
Source: J Clin Med. 2020 Nov 19;9(11):3719. doi: 10.3390/jcm9113719 (PMC7699602; doi:10.3390/jcm9113719)
Supplement: Supplementary file 1 [file jcm-09-03719-s001.pdf]

Journal of
Clinical Medicine

MDPI

Supplementary Material

Operators: ^, "starting with"; ǀ, logical OR

Supplementary Table S1. Operationalization of the cardiovascular risk categories according to the 2016 European Society of Cardiology Guidelines. The implementation of the cardiovascular risk categories in the FIRE database is described in detail in Meier et al. (Meier, R.; Rachamin, Y.; Rosemann, T.; Markun, S. The Impact of the 2019 European Guideline for Cardiovascular Risk Management: A Cross-Sectional Study in General Practice. J. Clin. Med. 2020, 9, 2140).

ESC Risk Category	Operationalization Based on Morbidities and SCORE
Very high risk	Atherosclerotic cardiovascular disease ǀ diabetes mellitus with target organ damage ǀ diabetes mellitus with a major risk factor ǀ severe chronic kidney disease ǀ SCORE[1] ≥10%
High risk	single risk factors (very high blood pressure or cholesterol) ǀ diabetes mellitus without risk factors/target damage ǀ moderate chronic kidney disease ǀ SCORE[1]≥5% and <10%
Moderate risk	SCORE[1] ≥1 and <5
Low risk	SCORE[1] < 1

Abbreviations: SCORE, Systematic Coronary Risk Estimation. [1]The SCORE directly provides prognostic probabilities for fatal cardiovascular events based on gender, age (40–70 years), smoking status, parametric values of systolic blood pressure (120–180 mmHg) and total cholesterol (4–8 mmol/L) in untreated patients without cardiovascular disease.

Supplementary Table S2. Definition of morbidities.

Morbidities	Operationalization Based on ATC Code, ICPC-2 code, Laboratory Measurements or Vital Values
Atherosclerotic cardiovascular disease	Any of the following:<br>• ICPC-2 = K74 ǀ K75 ǀ K76 ǀ K89 ǀ K90 ǀ K91 ǀ K92<br>• ATC code B01AC04 ǀ B01AC22 ǀ B01AC25 ǀ B01AC24
Diabetes mellitus	Any of the following:<br>• ICPC-2 = T89 ǀ T90<br>• Glycated hemoglobin > 6.5%<br>• ATC code ^A10
Severe chronic kidney disease	Glomerular filtration rate < 30 mL/min/1.73 m²
Moderate chronic kidney disease	Glomerular filtration rate <60 and ≥30 mL/min/1.73 m²
Hypertension	Any of the following:<br>• ICPC-2 = K85 ǀ K86 ǀ K87<br>• two blood pressure measurements > 140/90 mmHg<br>• ATC code ^C02 ǀ ^C03A ǀ C03EA01 ǀ ^C07 ǀ ^C08 ǀ ^C09A ǀ ^C09B
Obesity	Any of the following:<br>• ICPC-2 = T82<br>• Body mass index > 30 kg/m²

Abbreviations: ATC, Anatomical Therapeutic Chemical; ICPC-2, International Classification of Primary Care 2nd edition.

Supplementary Table S3. Description of patients for whom statin initiation is recommended, stratified by guideline concordance of the treatment decision.

Variable	Concordant (n = 531)	Undertreatment (n = 2882)
Median deviation of LDL-C from treatment thresholds (IQR) in mmol/L	1.9 (1.1–2.8)	1.3 (0.7–2.0)
% at very high risk (vs. high risk)	61.8	53.5
% female	48.2	49.2
Median age at index (IQR)	67 (59–73)	67 (58–75)
Continuity of care		

% COCI = 1	65.2	65.8
% COCI < 1	27.7	33.0
% missings[1]	7.2	1.2
GP characteristics		
% female GPs	22.2	13.6
Median age of GPs (IQR)	51 (43–59)	54 (47–60)

Abbreviations: LDL-C, low-density lipoprotein cholesterol; IQR, interquartile range; CV, cardiovascular; COCI, continuity of care index; GP, general practitioner. [1] For patients with only one consultation, no COCI could be calculated.
